# A conceptual framework for interventions targeting stigma associated with academic failure among repeating nursing students in nursing education

**DOI:** 10.4102/curationis.v49i1.2904

**Published:** 2026-10-30

**Authors:** Esther A. Mpangane, Nkhensani F. Mabunda, Deliwe R. Phetlhu

**Affiliations:** 1 Department of Nursing, Faculty of Health Care Sciences, Sefako Makgatho Health Sciences University, Tshwane, South Africa; 2 Department of Nursing, Faculty of Health Science, University of the Free State, Bloemfontein, South Africa

**Keywords:** stigma, repeating nursing students, non-repeating nursing students, nurse educators, nursing education institutions, conceptual framework

## Abstract

**Background:**

Academic failure among nursing students remains a significant challenge within nursing education institutions, often accompanied by stigma associated with academic failure (SAWAF). The SAWAF contributes to adverse psychological, social and academic consequences among repeating nursing students (RNS), potentially affecting their academic progression and professional identity formation. Despite increasing recognition of this issue, limited attention has been given to the systemic and cyclical nature of stigma-structured interventions aimed at addressing stigma within nursing education.

**Objectives:**

To develop a conceptual framework that explains and addresses the cyclical nature of SAWAF in nursing education.

**Methods:**

Qualitative descriptive design using triangulation. Data were drawn from three qualitative datasets exploring the experiences of RNSs, perceptions of nurse educators (NEs) and non-repeating nursing students. A triangulation approach was used to integrate findings and identify patterns of convergence and divergence. Thematic synthesis informed the development of a conceptual framework comprising root causes, key drivers, impacts and intervention points.

**Results:**

The findings revealed that SAWAF is sustained through interconnected institutional policies, NE behaviours and peer dynamics, which collectively contribute to psychological distress, academic underperformance, social exclusion and financial hardship among RNS. The proposed framework highlights NEs’ training, equitable teaching practices and institutional policy reforms as critical for disrupting the cycle.

**Conclusion:**

The proposed framework provides an evidence-informed model to guide interventions aimed at reducing stigma, strengthening educator practices and fostering inclusive learning environments in nursing education.

**Contribution:**

This study contributes an evidence-informed conceptual framework integrating institutional, educator and peer-level influences on SAWAF and identifying intervention points to disrupt its cyclical reproduction in nursing education.

## Introduction

Stigma associated with academic failure (SAWAF) represents a significant yet insufficiently addressed challenge within nursing education. While academic failure is often conceptualised as an individual deficit, emerging evidence suggests that it is deeply embedded within institutional, interpersonal and social contexts (Lewis 2020; Mpangane, Mabunda & Phetlhu 2026).

These findings align with broader literature demonstrating that stigma is socially constructed and reinforced within educational and institutional environments (Nyblade et al. 2019). Academic failure in nursing education has been widely associated with progression delays, attrition and compromised professional development among students (Pierre 2023; Tonelli 2022). Repeating nursing students (RNS) frequently encounter labelling, exclusion and diminished academic identity, which collectively compromise their learning experiences and professional development (Mpangane et al. 2026).

Similar findings have been reported internationally, where nursing students who experience academic failure often encounter social exclusion, diminished self-worth, psychological distress and reduced academic engagement (Lewis 2020; Pierre 2023; Tonelli 2022). These experiences are reinforced by institutional structures and interpersonal interactions that contribute to the social construction and perpetuation of stigma within educational environments (Nyblade et al. 2019).

Within nursing education institutions (NEIs), SAWAF is reinforced through policies, nurse educators’ (NEs’) practices and peer interactions. These factors not only shape student experiences but also contribute to a cycle in which academic failure and stigma mutually reinforce one another. Stigma associated with academic failure is increasingly recognised as a barrier to learning, reinforcing negative self-perceptions and limiting RNS engagement (Nunes et al. 2022; Wahid et al. 2024). Consequently, interventions that focus solely on individual remediation are insufficient (Mpangane et al. 2026).

Although previous studies have documented the consequences of academic failure among nursing students, limited research has examined how SAWAF is produced, sustained and potentially disrupted within nursing education systems.

This study forms part of a broader doctoral research study. It builds on Phase One findings that integrated the perspectives of RNS, NEs and non-repeating nursing students (NRNS), which revealed SAWAF as a systemic and cyclical phenomenon.

Conceptual frameworks play a critical role in translating complex qualitative findings into structured, actionable models (Ojika et al. 2024). By integrating multiple perspectives, such frameworks enable the identification of systemic drivers and inform targeted, evidence-based interventions. This study, therefore, aims to develop a conceptual framework that explains the cyclical nature of SAWAF and identifies key intervention points in nursing education.

Although the framework is primarily derived from empirical findings of this study, it is conceptually informed by existing literature on stigma, educational systems and student experiences of academic failure.

Although SAWAF has increasingly been recognised as an important challenge in nursing education, there remains limited theoretical and practical guidance for understanding how institutional structures, NE practices and peer dynamics interact to sustain this phenomenon. Furthermore, no evidence-informed conceptual framework has been developed to integrate these interconnected influences and guide intervention development within nursing education institutions. Building on empirical findings generated through a multi-phase doctoral study involving RNS, NEs and NRNS, this study addresses this gap by developing an evidence-informed conceptual framework that explains the cyclical nature of SAWAF, identifies its principal drivers and consequences, and proposes key intervention points to inform nursing education practice, institutional policy and future research.

## Aim

To develop a conceptual framework that explains and addresses the cyclical nature of SAWAF in nursing education.

## Research methods and design

### Design

This study formed part of a broader multi-phase doctoral research programme investigating SAWAF among RNS in nursing education. Phase One generated qualitative evidence by exploring the experiences and perceptions of RNS, NEs and NRNS regarding SAWAF. Phase Two involved the integration and triangulation of these findings to identify areas of convergence, complementarity and divergence across stakeholder groups. Building on these empirical findings, the present phase employed a qualitative descriptive framework development design to synthesise the evidence into an evidence-informed conceptual framework for guiding interventions aimed at reducing SAWAF in nursing education.

### Setting

The study was conducted across four campuses of a nursing education institution in South Africa. These campuses provide the R.171 Diploma in Nursing programme and are characterised by diverse student populations and shared institutional policies governing academic progression, assessment and student support. The institutional context is relevant as it reflects common structural and academic challenges within South African nursing education.

### Participants

The datasets were derived from Phase One of the doctoral study, which explored SAWAF from three main stakeholder perspectives: RNS, NEs and NRNS.

### Data collection

Data were derived from three qualitative groups generated during Phase One of a broader doctoral study. Data collection methods included individual semi-structured interviews with RNS and focus group discussions with NEs and NRNS. Interview guides were designed to explore experiences and perceptions related to SAWAF.

### Data analysis and triangulation

Data were analysed using thematic analysis. Initial coding was conducted within each dataset, followed by cross-case comparison to identify converging and diverging patterns. Themes were then abstracted into higher-order categories representing root causes, drivers, impacts and intervention points. Subsequently, a triangulation process was applied to compare and integrate findings across the datasets. This involved systematic identification of areas of convergence (shared patterns), complementarity (expanding insights) and divergence (contrasting perspectives). An iterative analytical process was used to construct the conceptual framework, emphasising relationships and feedback loops within SAWAF.

### Trustworthiness

Trustworthiness was established using Lincoln and Guba’s criteria of credibility, dependability, confirmability and transferability.

Credibility was enhanced through methodological triangulation and the integration of three complementary qualitative datasets involving RNS, NEs and NRNS. Prolonged engagement with the datasets and iterative comparison of findings across stakeholder groups strengthened confidence in the consistency and authenticity of the emerging conceptual categories.

Dependability was ensured by maintaining a comprehensive audit trail documenting methodological decisions, coding procedures, thematic synthesis, triangulation processes and framework development. Regular supervisory consultations were conducted throughout the analytical process to ensure consistency and transparency.

Confirmability was strengthened through reflexive discussions between the primary researcher and supervisors, together with continual reference to the original qualitative data to ensure that the conceptual framework remained grounded in participants’ experiences rather than researcher assumptions. The development of the framework involved repeated movement between the original datasets, emerging analytical categories and relevant literature to verify the coherence of interpretations.

Transferability was supported through detailed descriptions of the study context, participant groups, data sources, analytical procedures and the conceptual framework development process, thereby enabling readers to determine the applicability of the findings to similar nursing education settings.

### Ethical considerations

Ethical approval was obtained from the relevant Research Ethics Committee and National Health Research Database. Informed consent was obtained from all participants. Anonymity and confidentiality were maintained throughout the study.

The study adhered to the Declaration of Helsinki for research involving human participants in health sciences. Ethical clearance to conduct this study was obtained from the Sefako Makgatho University Research Ethics Committee (No. SMUREC/H/486/2023: PG). Further approval was obtained from the National Health Research Database (GP202402115) to conduct the nursing education research.

### Conceptual framework development

The conceptual framework was developed through an iterative process of thematic synthesis, abstraction and conceptual integration using the triangulated findings generated during the preceding phase of the doctoral study. The process was informed by qualitative framework development approaches that advocate moving systematically from empirical findings to conceptual explanation.

Initially, the themes identified within each stakeholder dataset (RNS, NEs and NRNS) were compared to identify areas of convergence, complementarity and divergence. Similar concepts were grouped through constant comparison and progressively abstracted into higher-order conceptual categories representing the principal components of SAWAF.

Four interrelated analytical domains emerged from this process: root causes, key drivers, impacts and intervention points. Relationships among these domains were then examined iteratively to identify causal pathways, reciprocal influences and reinforcing feedback mechanisms. Rather than representing isolated categories, the analysis demonstrated that institutional structures, NEs’ practices, peer dynamics and student experiences interacted continuously to perpetuate SAWAF.

The emerging framework underwent repeated refinement through movement between the original qualitative datasets, thematic interpretations, supervisory discussions and relevant literature to ensure conceptual coherence and empirical grounding. This iterative process enabled the development of a framework that remained firmly anchored in participants’ experiences while extending beyond descriptive findings to provide an explanatory model.

The final conceptual framework (Figure 1) conceptualises SAWAF as a dynamic, cyclical and self-reinforcing process. It illustrates how institutional policies, NEs’ practices and peer interactions collectively contribute to the production and maintenance of stigma, while identifying evidence-informed intervention points capable of disrupting this cycle and promoting inclusive nursing education environments.

**FIGURE 1:**
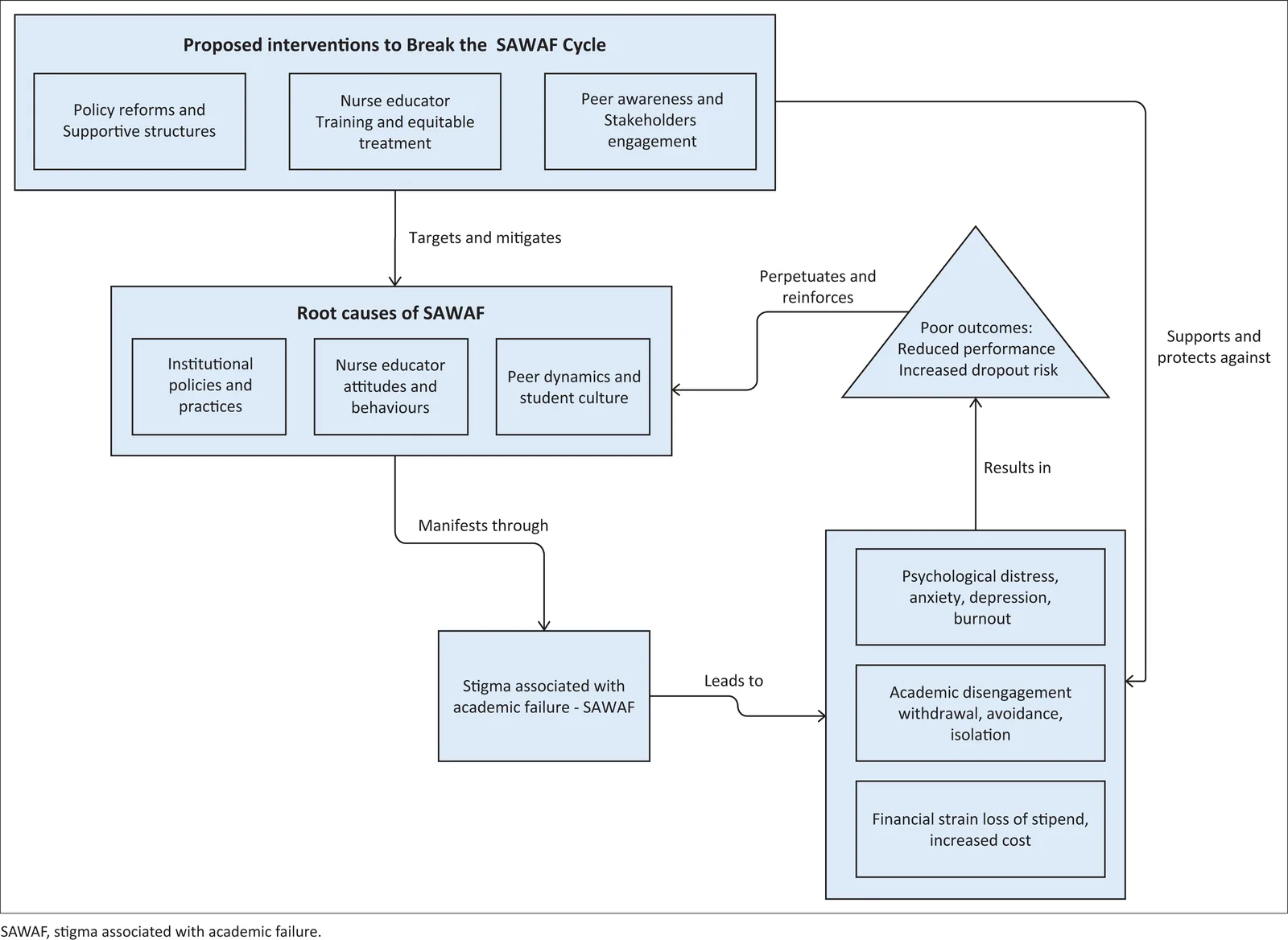
Conceptual framework for interventions targeting stigma associated with academic failure among repeating nursing students.

## Results

### Stigma associated with academic failure as a cyclical phenomenon

The integrated findings demonstrate that SAWAF is a cyclical process sustained by interconnected institutional, interpersonal and social factors. Structural conditions within NEIs create environments in which SAWAF is produced, while NEs’ practices and peer interactions reinforce and perpetuate these conditions. This cyclical interaction results in the ongoing stigmatisation of RNS.

### Root causes and drivers of the stigma associated with academic failure cycle

The findings indicate that SAWAF is sustained through the interaction between underlying structural conditions and the mechanisms through which these conditions are enacted. Three primary root causes were identified as institutional policies, NEs’ behaviours and peer dynamics. These root causes create the context within which SAWAF emerges, while specific practices function as drivers that actively reproduce SAWAF within NEIs.

Institutional policies constitute a central root cause shaping RNS’ experiences of academic failure. Administrative practices such as publicly identifying nursing students who repeat modules and withdrawal of financial support create visible distinctions between RNS and their NRNS peers. These root causes are operationalised through key drivers, including labelling practices and the withdrawal of financial support, both of which reinforce negative experiences and perceptions of academic failure and position RNS as stigmatised within the academic environment.

Nurse educators’ behaviours further contribute to producing and reinforcing SAWAF. While NEs often intend to support academically struggling students, certain practices inadvertently function as drivers of SAWAF. These include publicly identifying RNS, applying differential treatment and demonstrating unprofessional conduct, such as breaches of confidentiality and high expectations. Such practices not only reinforce SAWAF but also shape classroom dynamics in ways that undermine inclusivity.

Peer dynamics represent an additional root cause, particularly within competitive academic environments. The tendency of NRNS to distance themselves from RNS creates conditions for social exclusion. This exclusion is enacted through specific drivers, including limited collaboration, questioning of academic credibility and reduced peer support. These processes reinforce negative stereotypes and contribute to the social stigmatisation of RNS.

The interaction between root causes and drivers highlights that SAWAF is not produced by isolated practices but emerges from interconnected processes that continuously reinforce SAWAF within the NEIs environment.

### Impacts of stigma associated with academic failure

The impacts of SAWAF were evident across psychological, academic, social and financial domains. Repeating nursing students reported emotional distress, including anxiety, reduced self-confidence and feelings of embarrassment, which negatively influenced academic engagement. These psychological consequences are well documented in studies linking stigma and academic failure to decreased self-efficacy and increased anxiety among nursing students (Nunes et al. 2022; Wahid et al. 2024).

Socially, SAWAF contributed to isolation and limited peer interaction, while financial challenges further constrained nursing students’ ability to participate fully in academic activities. These combined effects often resulted in reduced motivation and increased risk of continued academic difficulty among RNS.

Importantly, these impacts reinforce the cycle of SAWAF by perpetuating negative self-perceptions and limiting opportunities for recovery, thereby feeding back into the structural and interpersonal processes that sustain SAWAF.

### Interventions to disrupt the cycle

The conceptual framework identifies three key intervention domains aimed at disrupting the SAWAF cycle. Nurse educator training emerged as the central intervention point, given the influential role of NEs in shaping learning environments. Training that promotes SAWAF awareness, ethical conduct and inclusive teaching practices may reduce stigmatising behaviours. Institutional policy reform represents a structural intervention domain. Revising policies that publicly identify students or impose disproportionate financial support may reduce structural drivers of stigma.

Peer engagement strategies, including mentorship and collaborative learning initiatives, may address social exclusion and promote supportive nursing student relationships. These interventions operate at multiple levels and collectively provide a comprehensive approach to disrupting the SAWAF cycle.

### Conceptual framework visualisation

The findings were synthesised into a conceptual framework (Figure 1), which visually illustrates the cyclical nature of SAWAF and the relationships between root causes, key drivers, impacts and intervention points. The framework demonstrates how institutional structures, NE practices and peer interactions interact to sustain SAWAF, while highlighting opportunities for intervention. The framework provides a visual synthesis of the integrated findings and serves as the foundation for the discussion of how SAWAF is produced, sustained and can be disrupted within nursing education environments.

## Discussion

The conceptual framework developed in this study highlights the interconnected nature of institutional, interpersonal and social influences on SAWAF. Drawing on triangulated findings from a broader doctoral study, this study conceptualises SAWAF as a systemic and cyclical phenomenon embedded within NEIs. The findings demonstrate that SAWAF is not merely an individual experience of academic difficulty but is produced and sustained through the interaction of institutional policies, NEs’ practices and peer dynamics. This extends existing literature by illustrating how SAWAF is structurally reproduced through routine educational practices and organisational systems, reinforcing inequities in RNS experiences and outcomes.

At the institutional level, policies and administrative processes emerged as foundational drivers of SAWAF. Practices such as the public identification of RNS, inconsistent implementation of academic policies, and the withdrawal of financial support institutionalised SAWAF within institutional structures. These processes transform academic failure into a visible and enduring identity marker, reinforcing stigmatisation and limiting opportunities for academic recovery. This supports evidence that institutional practices and structural conditions play a central role in producing and sustaining stigma within healthcare and educational systems (Nyblade et al. 2019).

These findings support Nyblade et al. (2019), who argue that stigma is frequently embedded within institutional systems and routine organisational practices that normalise exclusion and reinforce disadvantage. Similarly, Lewis (2020) and Pierre (2023) demonstrated that institutional responses to academic failure substantially influence nursing students’ educational experiences and progression. From a broader theoretical perspective, Andersen, Varga & Folker (2022) and Rodat (2020) contend that stigma is sustained through social and structural processes that legitimise separation, status loss and unequal treatment rather than through individual attitudes alone.

The findings suggest that institutional systems do not merely reflect SAWAF but actively produce and legitimise it through routine practices. This aligns with conceptualisations of SAWAF as structurally embedded within organisational environments rather than solely enacted through interpersonal interactions.

The implications for institutional practice are therefore significant. Addressing SAWAF requires a shift from deficit-oriented interpretations of academic failure towards a developmental and systems-based perspective. Institutional policies governing academic progression, assessment, and nursing student funding should be critically reviewed to ensure that they do not inadvertently perpetuate SAWAF. Transparent, equitable and supportive policy frameworks are essential for disrupting structural drivers of SAWAF. Without such reform, interventions targeting individual behaviours are unlikely to achieve sustained impact, as the broader system continues to reinforce stigmatising conditions.

At the interpersonal level, the role of NEs is central to both the perpetuation and disruption of SAWAF. The findings indicate that NEs may unintentionally reinforce SAWAF through practices such as public questioning, differential expectations and breaches of confidentiality. Although these behaviours are often framed as efforts to promote RNS accountability, they contribute to negative identity construction among RNS. This highlights the complexity of NE–student interactions, where well-intentioned practices may produce unintended consequences.

These findings are consistent with studies demonstrating that educator behaviours strongly influence nursing students’ sense of belonging, confidence, professional identity and willingness to seek support following academic failure (Elmir et al. 2019; Lewis 2020; Pierre 2023). Similar patterns of labelling, unequal treatment and public identification have also been reported within nursing education environments (Hadian Jazi et al. 2022; Richards, O’Connell & Dickinson 2023).

Consistent with the ADDIE–Lewin framework applied in the doctoral research study, these findings reinforce the importance of targeting NEs’ behaviour as a key entry point for change. The transformation of NEs’ practices requires more than awareness; it necessitates structured interventions that facilitate behavioural and attitudinal change. Professional development initiatives should therefore focus on promoting reflective teaching practices, ethical conduct and awareness of implicit bias. By equipping NEs with the skills to recognise and address stigmatising behaviours, such interventions can reposition NEs as agents of change within the nursing education system.

Embedding anti-stigma awareness within nursing curricula aligns with transformative learning principles, which emphasise critical reflection as a mechanism for challenging assumptions and changing professional practice (Mezirow 2000). Such approaches have been associated with improved inclusivity, empathy and learner-centred educational environments (Hadian Jazi et al. 2022; Richards et al. 2023). Integrating these principles into NE preparation and continuing professional development may therefore facilitate more inclusive educational practices while reducing stigmatising interactions with RNS.

From an educational perspective, these findings underscore the need to integrate anti-stigma principles within curriculum design and NEs preparation. Nursing education programmes should move beyond knowledge transmission to incorporate critical reflection on power dynamics, professional conduct and nursing student support. This approach aligns with transformative learning principles, which emphasise the role of reflection in reshaping deeply held beliefs and practices. Embedding SAWAF awareness within curricula may enhance both NEs’ responsiveness and RNS resilience, thereby contributing to more inclusive learning environments.

Peer dynamics further reinforce the cyclical nature of SAWAF. Similar findings have been reported across nursing and higher education contexts, where students who experience academic failure frequently describe peer distancing, reduced collaboration, diminished belonging and reluctance to seek academic support (Elmir et al. 2019; Hadian Jazi et al. 2022; Lewis 2020; Nunes et al. 2022; Richards et al. 2023). These observations are consistent with contemporary conceptualisations of stigma, which emphasise that labelling, social separation and status loss are sustained through interpersonal interactions that reinforce perceptions of difference and exclusion (Andersen et al. 2022; Rodat 2020). In the present study, peer interactions extended beyond individual attitudes to reinforce the social identity of being a ‘repeater’, further undermining participants’ sense of belonging within the nursing education environment.

The cyclical interaction between perceived incompetence and social exclusion illustrates how SAWAF is sustained through everyday peer interactions.

Addressing this dimension requires targeted strategies to promote inclusive peer engagement. Structured initiatives such as peer mentorship, collaborative learning models and facilitated group integration may help to reduce SAWAF and foster supportive relationships among nursing students. These approaches should be embedded within teaching and learning practices to ensure that inclusion is actively promoted rather than assumed. By strengthening peer support systems, NEIs can reduce the social drivers of SAWAF and enhance nursing student engagement.

The conceptual framework represents the principal theoretical contribution of this study. Rather than viewing SAWAF as an isolated interpersonal phenomenon, the framework conceptualises it as a dynamic system in which institutional structures, NE practices, peer interactions, and students’ psychological responses interact through reciprocal feedback mechanisms. This systems perspective extends previous work on academic failure, which has largely focused on predictors of failure or student remediation (Lewis 2020; Pierre 2023), by illustrating how stigma is continuously reproduced within nursing education institutions.

The present findings are also consistent with Tonelli (2022) and Pierre (2023), who highlighted that the consequences of course repetition extend beyond academic performance and encompass emotional, social, and professional identity challenges. These findings reinforce the need for comprehensive interventions that address both individual experiences and systemic contributors to academic failure stigma.

Importantly, the framework identifies NEs training as a central and immediate intervention point, while recognising that sustainable change requires alignment with institutional policy reform and supportive peer environments. This multi-level approach reflects best practices in educational intervention design, where behavioural change must be supported by enabling structures and cultures. The alignment of individual, interpersonal and organisational interventions enhances the potential for long-term impact and systemic transformation.

The conceptual framework provides an evidence-informed foundation for designing multi-level interventions that simultaneously target institutional systems, NE practices and student support. Such approaches have been recommended as the most effective means of addressing complex educational stigma because they address both structural and interpersonal determinants of student experiences (Nyblade et al. 2019; Pierre 2023; Tonelli 2022). Consequently, the framework offers practical guidance for improving teaching practices, strengthening student support systems, promoting inclusive learning environments, and enhancing student well-being, engagement, retention and academic success within nursing education institutions.

At the policy level, the findings emphasise the need for systemic reform to address the structural determinants of SAWAF. Institutional leadership plays a critical role in driving this transformation by promoting equitable practices and embedding anti-SAWAF principles within organisational culture. Policies that publicly label RNS or impose disproportionate support for academic failure should be reconsidered, as they contribute to the perpetuation of SAWAF. The integration of anti-SAWAF principles within NEIs frameworks is essential for ensuring that interventions are sustained over time.

Consistent with prior research on academic failure and course repetition, these findings highlight that student outcomes are shaped not only by individual performance but also by systemic and contextual factors within nursing education (Pierre 2023; Tonelli 2022). Overall, the findings demonstrate that SAWAF is not an isolated phenomenon but a socially constructed and institutionally reinforced process embedded within nursing education systems. The cyclical nature of SAWAF underscores the need for coordinated interventions that address multiple levels of influence, including institutional structures, NEs’ practices and peer interactions. By integrating these dimensions, the study contributes a comprehensive understanding of SAWAF and provides a foundation for the development of effective and sustainable interventions.

Institutional leadership plays a pivotal role in disrupting the structural drivers of stigma through equitable policies, transparent academic procedures and supportive organisational cultures. Similar recommendations have emerged from international work demonstrating that sustainable reductions in educational stigma require institutional commitment rather than isolated educational interventions (Lewis 2020; Nyblade et al. 2019; Pierre 2023).

Collectively, these findings extend existing scholarship by demonstrating that SAWAF is simultaneously structural, interpersonal, social and psychological. The study therefore moves beyond descriptive accounts of student failure to provide an integrated conceptual explanation of how stigma is produced, reinforced and potentially disrupted within nursing education institutions. This conceptual advancement provides an evidence-informed foundation for the design, implementation and evaluation of multi-level interventions aimed at reducing SAWAF and promoting equitable, inclusive nursing education environments.

### Strengths and limitations

A major strength of this study is the triangulation of perspectives from RNS, NEs and NRNS, which enhanced the depth, credibility and comprehensiveness of the findings. Furthermore, the framework was derived from empirical evidence generated through a multi-phase doctoral study, providing a robust foundation for intervention development. The inclusion of a visual conceptual framework offers a practical tool for guiding educational, institutional and policy interventions aimed at reducing SAWAF within nursing education.

### Limitations

This study was conducted within a single nursing education institution, which may limit the transferability of the findings to other educational contexts. Although the triangulation of perspectives from RNS, NEs and NRNS enhanced the credibility and depth of the findings, the study relied on secondary analysis of existing qualitative datasets. Consequently, opportunities to explore emerging issues through additional data collection were limited. Furthermore, the conceptual framework has not yet been empirically tested in practice, and its effectiveness in reducing SAWAF requires further evaluation across diverse nursing education settings.

### Recommendations for practice, nursing education, policy and research

Based on the findings of this study, nursing education institutions should implement coordinated multi-level interventions targeting institutional systems, NE practices and peer learning environments to reduce SAWAF. Nurse educators should receive ongoing professional development focusing on SAWAF awareness, inclusive teaching practices, ethical conduct, confidentiality and equitable student support. Nursing education curricula should incorporate concepts related to stigma, equity, inclusivity and student well-being to promote reflective teaching and learning practices. Institutional policies governing academic progression, financial support and the management of RNS should be reviewed to ensure that they do not inadvertently reinforce stigma or social exclusion.

In addition, structured peer-support initiatives, mentorship programmes and collaborative learning opportunities should be implemented to strengthen social integration and reduce negative stereotyping. Future research should focus on implementing and evaluating the proposed conceptual framework across diverse nursing education institution contexts to determine its effectiveness in reducing SAWAF, improving student experiences and enhancing academic success and retention.

## Conclusion

Stigma associated with academic failure is not merely an individual student experience but a systemic and self-reinforcing phenomenon embedded within nursing education structures and practices. This study developed a conceptual framework that elucidates the cyclical nature of SAWAF and identifies key intervention points. By emphasising NE training, institutional reform, and peer engagement, the framework provides a practical foundation for disrupting SAWAF and promoting inclusive learning environments. Future research should implement and evaluate the framework across diverse nursing education settings to determine its effectiveness, scalability and sustainability in reducing SAWAF.
